# Maternal Morbidity and Birth Satisfaction After Implementation of a Validated Calculator to Predict Cesarean Delivery During Labor Induction

**DOI:** 10.1001/jamanetworkopen.2020.25582

**Published:** 2020-11-13

**Authors:** Rebecca F. Hamm, Jennifer McCoy, Amal Oladuja, Hilary R. Bogner, Michal A. Elovitz, Knashawn H. Morales, Sindhu K. Srinivas, Lisa D. Levine

**Affiliations:** 1 Maternal and Child Health Research Center, Department of Obstetrics and Gynecology, University of Pennsylvania Perelman School of Medicine, Philadelphia; 2 Department of Family Medicine and Community Health, University of Pennsylvania Perelman School of Medicine, Philadelphia; 3 Department of Biostatistics, Epidemiology and Informatics, University of Pennsylvania Perelman School of Medicine, Philadelphia

## Abstract

**Importance:**

A previously created and validated calculator provides an individualized cesarean delivery risk score for women undergoing labor induction. A higher predicted risk of cesarean delivery on the calculator has been associated with increased maternal and neonatal morbidity regardless of ultimate delivery mode. The effect of this calculator when implemented in clinical care has yet to be evaluated.

**Objective:**

To determine whether implementation of a validated calculator that predicts the likelihood of cesarean delivery at the time of labor induction is associated with maternal morbidity and birth satisfaction.

**Design, Setting, and Participants:**

This prospective cohort study used medical record review to compare the 1 year before calculator implementation (July 1, 2017, to June 30, 2018) with the 1 year after implementation (July 1, 2018, to June 30, 2019) at a US urban, university labor unit. Women admitted for labor induction with singleton gestation in cephalic presentation, intact membranes, and an unfavorable cervix were included. Data were analyzed from August 1, 2019, to September 13, 2020.

**Exposures:**

Patient and clinician knowledge of the calculated cesarean delivery risk score based on the validated calculator.

**Main Outcomes and Measures:**

The primary outcomes were (1) composite maternal morbidity defined by at least 1 of the following within 30 days of delivery: endometritis, postpartum hemorrhage (estimated or quantitative blood loss >1000 mL), blood transfusion, wound infection, venous thromboembolism, hysterectomy, intensive care unit admission, and readmission and (2) patient satisfaction assessed via Birth Satisfaction Scale–Revised (BSS-R) scores. Secondary outcomes included rate of cesarean delivery and neonatal morbidity.

**Results:**

A total of 1610 women were included in the analysis (788 in the preimplementation and 822 in the postimplementation periods) with a median age of 29 (interquartile range [IQR], 24-34) years. There were no significant baseline differences between groups except fewer inductions at a gestational age of 40 weeks or later in the postimplementation period (256 [31.1%] vs 298 [37.8%]). Calculator implementation was associated with decreased maternal morbidity overall, even when adjusting for confounders (141 [17.9%] vs 95 [11.6%]; adjusted absolute risk difference [aARD], −6.3%; 95% CI, −9.7% to −2.8%). Although there was no difference in birth satisfaction overall, calculator implementation was associated with improvements on items pertaining to quality of care provision (median BSS-R score, 19 [IQR, 16-20] vs 19 [IQR, 17-20]; *P* = .006). Calculator implementation was also associated with a decrease in cesarean delivery rate (228 [28.9%] vs 167 [20.3%]; aARD, −8.5% [95% CI, −12.6% to −4.5%]). There were no significant differences in neonatal morbidity.

**Conclusions and Relevance:**

These findings suggest that implementation of a validated calculator to predict risk of cesarean delivery in clinical care is associated with reduced maternal morbidity. Implementation should occur broadly to determine whether calculator use improves national maternal outcomes.

## Key Points

**Question:**

Is implementation of a validated calculator to predict cesarean delivery after labor induction associated with maternal morbidity and birth satisfaction?

**Findings:**

In this cohort study of 1610 women admitted for labor induction, use of a cesarean delivery risk calculator was associated with a 6% absolute risk reduction in maternal morbidity, as well as an 8% absolute risk reduction in cesarean delivery. Calculator implementation was associated with improvements in women’s perception of quality of care received.

**Meaning:**

These findings suggest that implementation of a validated calculator to predict risk of cesarean delivery in clinical care is associated with reduced obstetric morbidity and improved the patient birth experience.

## Teaser

This cohort study assesses whether implementation of a validated calculator for likelihood of cesarean delivery at the time of labor induction is associated with maternal morbidity and birth satisfaction.

## Introduction

Labor induction is the process of stimulating uterine contractions during pregnancy before labor begins on its own, with the goal of a vaginal birth. In the US, more than 4 million women give birth annually, with more than 20% undergoing an induction.^1^ Despite the fact that approximately one-third of inductions will end in a cesarean delivery, our ability to predict who will have a successful induction resulting in a vaginal birth is limited.^2,3,4^ In answer, Levine et al^5^ created a risk prediction model for cesarean delivery among women undergoing an induction that was both internally and externally validated in a database of more than 200 000 deliveries across 19 diverse hospitals with an area under the curve of 0.73. The risk prediction model was developed into a user-friendly online calculator (hereinafter referred to as the cesarean risk calculator) requiring only 5 pieces of data: height (in centimeters), body mass index (calculated as weight in kilograms divided by height in meters squared), parity, gestational age at the time of induction, and cervical examination findings. These items are input directly into the calculator at induction start, producing an individualized percentage risk of cesarean delivery for a patient’s induction.

When stratified into categories of cesarean delivery risk (<20.0%, 20.0%-39.9%, 40.0%-59.9%, and ≥60.0%), a higher predicted probability of cesarean delivery on this calculator is also associated with longer labor, as well as increased maternal morbidity (<20.0% risk, 2.6% probability; 20.0%-39.9% risk, 4.7% probability; 40.0%-59.9% risk, 5.1% probability; and ≥60.0% risk, 6.1% probability; *P* = .001) and neonatal morbidity (<20.0% risk, 0.9% probability; 20.0%-39.9% risk, 1.5% probability; 40.0%-59.9% risk, 2.0% probability; and ≥60.0% risk, 2.2% probability; *P* = .002).^6^ Thus, the higher the calculated risk of cesarean delivery, regardless of the actual mode of delivery, the higher the risk of an adverse outcome.

In addition, before development of this calculator, women and their clinicians began an induction without the knowledge of how likely the patient was to have a cesarean delivery. Improved prediction of cesarean delivery has the potential to improve patient satisfaction by clarifying patient expectations. Patient satisfaction during labor is directly linked to mother-infant bonding, breastfeeding rates, and decreased risk for postpartum depression.^7,8^

Whether implementation of this calculator into clinical care will affect perinatal outcomes is unknown. This study aimed to evaluate (1) maternal morbidity and (2) patient satisfaction before and after implementation of the cesarean risk calculator. We hypothesized that if a clinician is aware of a patient’s individualized risk of cesarean delivery, this knowledge will influence labor induction management, leading to a decrease in maternal morbidity without increasing the rate of cesarean delivery. In addition, we hypothesized that providing patients with the knowledge of their risk of cesarean delivery at the start of induction will improve satisfaction with the birth experience.

## Methods

We performed a prospective cohort study of women undergoing labor induction at the Hospital of the University of Pennsylvania, Philadelphia, before and after implementation of the cesarean risk calculator into usual clinical care. The preimplementation period was from July 1, 2017, to June 30, 2018. The calculator was implemented as standard of care on July 1, 2018, and the postimplementation period was from July 1, 2018, to June 30, 2019. The project was approved by the University of Pennsylvania institutional review board as quality improvement with a waiver of informed consent due to its nature as quality improvement. The Strengthening the Reporting of Observational Studies in Epidemiology (STROBE) reporting guidelines were followed in the writing of this report.^9^

### Study Population

Women were included in this study if they were undergoing a term (≥37 weeks) labor induction for any indication and met the following inclusion criteria: 18 years or older, singleton gestation in cephalic presentation, intact membranes, and an unfavorable cervix (Bishop score of ≤6 [range, 0-13, with higher scores indicating favorable cervical examination results] and cervical dilation ≤2 cm). Women were excluded from the study if they had a prior cesarean delivery; had a contraindication to vaginal delivery; had a major fetal anomaly; did not speak English; had HIV, hemolysis, elevated liver enzyme levels, and low platelet count (HELLP syndrome); had eclampsia; or had intrauterine growth restriction with abnormal umbilical artery Doppler findings.^5^ These were the same criteria used to derive and validate the calculator.

### Implementation of the Calculator

On July 1, 2018, the calculator was implemented as standard of care for women undergoing induction on our labor unit who met inclusion criteria. After the start of the postimplementation period, the admitting clinician obtained the calculator result and placed a sticker with the predicted likelihood of cesarean delivery next to the patient’s information on the central labor and delivery board, visible to all clinicians and nurses. The clinician then counseled the patient on their range of cesarean delivery risk (<20.0%, 20.0%-39.9%, 40.0%-59.9%, or ≥60%) with the assistance of premade, standardized scripts. Documentation of this counseling was placed in the electronic medical record. Clinical care occurred at the discretion of the covering labor floor clinicians with no dictated changes in labor management based on the calculator score. Implementation strategies to incorporate the induction calculator into care included (1) multidisciplinary stakeholder buy-in, (2) education and training sessions, and (3) daily, clinician-specific audit and feedback on appropriate calculator use.

### Outcomes

The first primary outcome was a composite maternal morbidity defined by 1 or more of the following within 30 days of delivery: endometritis, postpartum hemorrhage (estimated or quantitative blood loss >1000 mL), blood transfusion, wound infection, venous thromboembolism, hysterectomy, intensive care unit admission, and readmission. Our second primary outcome was patient satisfaction using the validated Birth Satisfaction Scale–Revised (BSS-R), administered to patients during their inpatient postpartum stay. The BSS-R has been demonstrated to be a robust, valid, and reliable multidimensional psychometric instrument for measuring postpartum women’s birth satisfaction in diverse populations (Cronbach α = 0.79).^10,11^ The BSS-R asks women to report agreement or disagreement with 10 statements using a 5-point Likert scale. The total BSS-R ranges from 10 to 50, with higher scores indicating increased satisfaction. The BSS-R is subdivided into 3 domains: (1) quality of care provision, (2) stress experienced during labor, and (3) women’s personal attributes.

Our secondary maternal outcomes included rate of cesarean delivery, time from induction to delivery, indication for cesarean delivery, chorioamnionitis, and maternal length of stay. Secondary neonatal outcomes included composite neonatal morbidity (defined by ≥1 of the following: supplemental oxygen for ≥12 hours, and culture-proven or presumed neonatal sepsis), neonatal intensive care unit admissions, and neonatal length of stay.

### Data Collection

Baseline demographic data, medical and obstetrical history, and labor and delivery data were collected on all patients meeting inclusion criteria. Self-reported race/ethnicity was collected, because significant racial/ethnic disparities exist in rates of our primary and secondary outcomes. The BSS-R surveys were administered for the latter 6 months of the preimplementation period and the full 12 months of the postimplementation period. In addition, frequency of documentation regarding counseling around the calculator result in the postimplementation period was collected.

### Sample Size

In prior work at our institution, approximately 750 women per year met our inclusion criteria,^5^ with a baseline maternal morbidity rate of 15%. To have greater than 80% power with α = .05 to detect a 35% reduction in maternal morbidity, this required a sample size of 1 year before and 1 year after implementation. In addition, using data from a 2016 study of 2229 US women surveyed using the 10-question BSS-R, the mean (SD) score was 31.94 (6.75).^5,10^ We therefore had greater than 90% power to detect a 10% increase in patient satisfaction with our proposed sample size.

### Statistical Analyses

Data were analyzed from August 1, 2019, to September 13, 2020. Bivariate comparisons of demographic and preinduction clinical characteristics by preimplementation and postimplementation groups, as well as labor and delivery outcomes, were performed with Fisher exact tests and χ^2^ tests for categorical variables and unpaired, 2-tailed *t* tests or Wilcoxon rank sum tests for continuous variables, where appropriate. For the outcomes of composite maternal morbidity and cesarean delivery rate, directed acyclic graphs were used to determine potential confounders. Although only gestational age at delivery and indication for induction differed by exposure (calculator implementation), other covariates that could affect both the decision to use the calculator outside of our setting, as well as the outcomes, were included (parity and maternal age). Multivariable logistic regression modeling was used to adjust for all confounders and calculate adjusted absolute risk difference (aARD). Of note, gestational age at delivery and indication for induction were collinear and, of the two, only gestational age was included in final models. Sensitivity analysis was performed evaluating only those women with documented calculator result counseling.

In addition, the association between exposure and outcome was stratified by 4 risk groups for cesarean delivery based on calculator results (<20.0%, 20.0%-39.9%, 40.0%-59.9%, and ≥60.0%). Calculations of cesarean delivery risk were made retrospectively for the preimplementation group and not available in real time. To examine the association of the cesarean risk calculator in individual predicted risk strata, the interaction between preimplementation and postimplementation periods and the predicted group for cesarean delivery risk was added to the above models. Strata-specific aARDs were then determined using the appropriate contrast of coefficients.

To evaluate birth satisfaction, total and individual domain BSS-R scores were compared using the Wilcoxon rank sum test. Linear regression compared BSS-R scores, including possible confounders as described above. Statistical analyses were performed with Stata, version 15 (StataCorp LLC). All tests were 2 tailed, and *P* < .05 was considered statistically significant.

## Results

A total of 1610 women during the study period from July 1, 2017, to June 30, 2019, met inclusion criteria. Our population had a median age of 29 (interquartile range [IQR], 24-34) years; 1045 were Black (64.9%); and 871 (54.1%) were insured through Medicaid. Seven hundred and eighty-eight were included in the preimplementation group; 822, in the postimplementation group. In the postimplementation group, patient counseling regarding the induction calculator result was documented in the electronic medical record in 676 of 822 women (82.2%).

Demographic and clinical characteristics are detailed in Table 1. There were no significant differences in age, race/ethnicity, insurance, body mass index, parity, or medical comorbidities between groups. Fewer inductions occurred at a gestational age of 40 weeks or more in the postimplementation group (298 [37.8%] vs 256 [31.1%]; *P* = .005). This finding was also reflected in the indication for induction, with more elective inductions at 39 weeks in the postimplementation period (129 [16.4%] vs 182 [22.1%]; *P* = .003) and fewer postdate inductions (90 [11.4%] vs 60 [7.3%]; *P* = .004). However, although gestational age was one of the calculator inputs, overall calculated cesarean delivery risk did not differ between groups.

**Table 1.** Demographic and Clinical Characteristics of the Preimplementation and Postimplementation Periods

Characteristic	Study period^a^		*P* value
	Preimplementation (n = 788)	Postimplementation (n = 822)	
Maternal age, median (IQR),	29 (24-34)	29 (24-33)	.88
Race/ethnicity			
Black	514 (65.2)	531 (64.6)	.67
White	177 (22.5)	201 (24.5)	
Asian	55 (7.0)	48 (5.8)	
Other	42 (5.3)	42 (5.1)	
Hispanic	30 (3.8)	39 (4.7)	.35
Insurance			
Private	349 (44.3)	362 (44.0)	.59
Medicaid/Medicare	428 (54.3)	443 (53.9)	
Uninsured	11 (1.4)	17 (2.1)	
Maternal BMI at last prenatal visit, median (IQR)	32 (28.2-37.6)	32.4 (27.8-37.9)	.95
Gestational diabetes	69 (8.8)	59 (7.2)	.24
Pregestational diabetes	14 (1.8)	26 (3.2)	.07
Chronic hypertension	70 (8.9)	75 (9.1)	.87
Nulliparity	497 (63.1)	515 (62.7)	.86
Gestational age ≥40 wk	298 (37.8)	256 (31.1)	.005
Indications for induction			
Postdate pregnancy	90 (11.4)	60 (7.3)	.001
Maternal^b^	284 (36.0)	316 (38.4)	
Fetal^c^	285 (36.2)	264 (32.1)	
Elective or other^d^	129 (16.4)	182 (22.1)	
Scheduled induction	416 (52.8)	461 (56.1)	.19
Cervical dilation at induction start, cm			
<1	249 (31.6)	253 (30.8)	.72
1-2	539 (68.4)	569 (69.2)	
Modified Bishop score, median (IQR)^e^	2 (1-3)	2 (1-3)	.25
Calculated cesarean delivery risk, %			
<20.0	274 (34.8)	266 (32.4)	.74
20.0-39.9	286 (36.3)	317 (38.6)	
40.0-59.9	174 (22.1)	182 (22.1)	
≥60.0	54 (6.9)	57 (6.9)	

Abbreviations: BMI, body mass index (calculated as weight in kilograms divided by height in meters squared); IQR, interquartile range.

^a^ This study sample includes all patients admitted for labor induction at the Hospital of the University of Pennsylvania from July 1, 2017, to June 30, 2019, meeting inclusion and exclusion criteria for use of the cesarean risk calculator. Unless otherwise indicated, data are expressed as number (percentage) of patients.

^b^ Examples include chronic hypertension, gestational hypertension, preeclampsia, diabetes, renal disease, history of venous thromboembolism, cardiac disease, or other chronic medical condition where induction was recommended.

^c^ Examples include oligohydramnios, intrauterine growth restriction, or abnormal results on fetal testing.

^d^ Examples include history of an intrauterine fetal demise, vaginal bleeding at term, or cholestasis.

^e^ Scores range from 0 to 13, with higher scores indicating a more favorable cervical examination finding.

For our primary outcome, calculator implementation was associated with overall decreased composite maternal morbidity from 141 of 788 (17.9%) to 95 of 822 (11.6%; *P* < .001) (Table 2). This risk difference held true when adjusting for gestational age at delivery, parity, and maternal age (aARD, −6.3%; 95% CI, −9.7% to −2.8%). Of the individual components of the maternal morbidity composite outcome, significant associated reductions were noted from the preimplementation to the postimplementation periods in the rates of postpartum hemorrhage (67 [8.5%] vs 43 [5.2%]; *P* = .009) and wound separation/infection (37 [4.7%] vs 11 [1.3%]; *P* < .001). In the secondary analysis, calculator implementation was also associated with decreased overall cesarean delivery rate, from 228 of 788 (28.9%) to 167 of 822 (20.3%; *P* < .001) (Table 3). Calculator implementation was associated with a more than 8% lower risk in cesarean delivery rate when adjusted for gestational age at delivery, parity, and maternal age (aARD, −8.5%; 95% CI, −12.6% to −4.5%). Sensitivity analysis including only those with documented counseling of cesarean delivery risk did not differ significantly from the primary analysis.

**Table 2.** Primary Composite Maternal Morbidity Outcome Compared Between Preimplementation and Postimplementation Periods

Outcome	Study period, No. (%) of patients		*P* value
	Preimplementation (n = 788)	Postimplementation (n = 822)	
Composite maternal morbidity	141 (17.9)	95 (11.6)	<.001
Components of composite maternal morbidity			
Postpartum hemorrhage^a^	67 (8.5)	43 (5.2)	.009
Blood transfusion	42 (5.3)	33 (4.0)	.21
Wound separation or infection	37 (4.7)	11 (1.3)	<.001
Readmission within 30 d	22 (2.8)	21 (2.6)	.77
Endometritis	22 (2.8)	14 (1.7)	.14
Venous thromboembolism	4 (0.5)	3 (0.4)	.72
Intensive care unit admission	3 (0.4)	6 (0.7)	.51
Hysterectomy	0	1 (0.1)	>.99

^a^ Defined by either estimated blood loss or quantitative blood loss of greater than 1000 mL.

**Table 3.** Maternal and Neonatal Secondary Outcomes Compared Between Preimplementation and Postimplementation Periods

Secondary outcomes	Study period^a^		*P* value
	Preimplementation (n = 788)	Postimplementation (n = 822)	
Cesarean delivery	228 (28.9)	167 (20.3)	<.001
Time from induction to cesarean delivery, median (IQR), h			
Overall	20.7 (14.9-27.3)	21.4 (14.6-28.5)	.61
Stratified by risk, %			
<20.0	16.4 (1.2-23.6)	13.1 (9.2-16.9)	.15
20.0-39.9	20.2 (15.5-26.7)	20.9 (15.7-27.9)	.64
40.0-59.9	20.5 (14.9-27.4)	23.9 (15.2-3.1)	.12
≥60.0	26.2 (19.1-29.4)	21.1 (16.3-25.1)	.05
Indication for cesarean delivery			
Failed induction	75 (32.9)	42 (25.1)	.15
Arrest of active phase	21 (9.2)	21 (12.6)	
Arrest of descent or failed operative delivery	28 (12.3)	14 (8.4)	
Nonreassuring fetal heart tones	90 (39.5)	73 (43.7)	
Elective or other	14 (6.1)	17 (10.2)	
Chorioamnionitis	122 (15.5)	100 (12.2)	.05
Maternal length of stay, median (IQR), d	3 (3-4)	3 (3-3)	<.001
Composite neonatal morbidity^b^	81 (10.3)	74 (9.0)	.39
NICU admission	65 (8.2)	71 (8.6)	.89
Neonatal length of stay, median (IQR), d	2 (2-2)	2 (2-3)	<.001

Abbreviations: IQR, interquartile range; NICU, neonatal intensive care unit.

^a^ Unless otherwise indicated, data are expressed as number (percentage) of patients.

^b^ Defined by at least 1 of the following: need for supplemental oxygen for at least 12 hours and culture-proven or presumed neonatal sepsis.

When stratified by calculated cesarean risk, calculator implementation was associated with a 6% absolute risk reduction in maternal morbidity in the group with predicted risk of cesarean delivery of less than 20.0% (35 [12.8%] vs 18 [6.8%]; aARD, −6.0% [95% CI, −11.0% to −1.0%]; *P* = .02) (Figure, A). Within this risk group, calculator implementation was also associated with a 6% absolute risk reduction in cesarean delivery (35 [12.8%] vs 17 [6.4%]; aARD, −6.4% [95% CI, −11.3% to −1.5%]; *P* = .01) (Figure, B). Notably, calculator implementation was associated with an elimination of cesarean deliveries for failed induction in this risk group (8 [2.9%] in the preimplementation group vs 0 in the postimplementation group).

**Figure.**
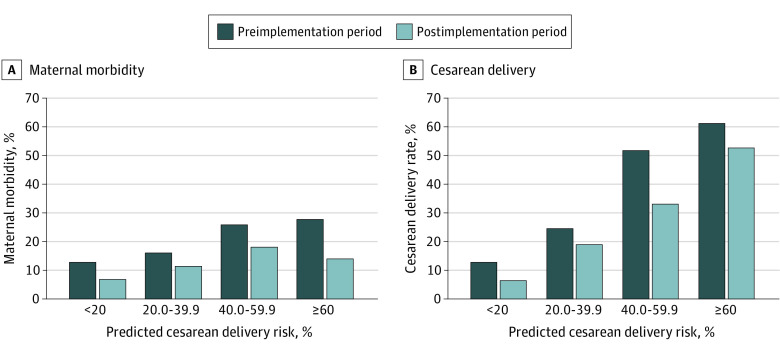
Adjusted Absolute Risk Differences in Outcomes Among Predicted Risk Groups for Cesarean Delivery Maternal morbidity and cesarean delivery are compared between the preimplementation and postimplementation periods.

No association of calculator implementation with absolute risk reduction was found in the group with predicted risk of cesarean delivery of 20.0% to 39.9% for maternal morbidity (46 [16.1%] vs 36 [11.4%]; aARD, −4.7% [95% CI, −10.2% to 0.8%]; *P* = .10) or cesarean delivery (70 [24.5%] vs 60 [18.9%]; aARD, −5.4% [95% CI, −12.0% to 1.2%]; *P* = .11). Within the group with predicted risk of cesarean delivery of 40.0% to 59.9%, calculator implementation was also associated with absolute risk reduction of 19% for cesarean delivery (90 [51.7%] vs 60 [33.0%]; aARD, −19.4% [95% CI, −29.4% to −9.3%]; *P* < .001) (Figure, B). A decrease in maternal morbidity was found, but it was not statistically significant (45 [25.9%] vs 33 [18.1%]; aARD, −7.8% [95% CI, −16.4% to 0.8%]; *P* = .07) (Figure, A).

In the group with predicted risk of cesarean delivery of 60.0% or greater, actual cesarean delivery rate after implementation did not change significantly (33 [61.1%] vs 30 [52.6%]; aARD, −9.6% [95% CI, −27.8% to 8.6%]; *P* = .30) (Figure, B). There was a decrease in maternal morbidity, but this finding was not statistically significant (15 [27.8%] vs 8 [14.0%]; aARD, −14.0% [95% CI, −28.9% to 1.0%]; *P* = .07) (Figure, A). A decrease in time to cesarean by more than 5 hours in this predicted cesarean delivery risk group also did not reach statistical significance (26.2 [IQR, 19.1-29.4] vs 21.1 [IQR, 16.3-25.1] hours; *P* = .05) (Table 3).

When comparing other secondary outcomes (Table 3), calculator implementation was associated with decreased median maternal length of stay (3 [IQR, 3-4] vs 3 [IQR, 3-3] days; *P* < .001). There were no differences in neonatal outcomes, although median neonatal length of stay was also shorter after implementation (2 [IQR, 2-3] vs 2 [IQR, 2-2] days; *P* < .001).

Three hundred and thirty of 414 eligible women (79.7%) completed the BSS-R in the 6 months before calculator implementation, and 678 of 822 (82.5%) completed it in the 1 year after implementation. Total and individual domain birth satisfaction scores are shown in Table 4. Although calculator implementation was associated with improved total birth satisfaction scores (39 [IQR, 34-42] vs 40 [IQR, 35-43]; *P* = .04) in bivariate analyses, there was no significant difference in total satisfaction scores in multivariable modeling (F_2,1005_ = 8.4; *R*^2^ = 0.02; *P* = .05). When analyzing the 3 BSS-R domains, calculator implementation was associated with improvement in quality of care provision (domain 2) (19 [IQR, 16-20] vs 19 [IQR, 17-20]; *P* = .006), even when controlling for possible confounders.

**Table 4.** Birth Satisfaction Scores as Determined by the BSS-R Compared Between Preimplementation and Postimplementation Periods

BSS-R component	Median (IQR) score by study period		*P* value
	Preimplementation (n = 330)	Postimplementation (n = 678)	
Total score^a^	39 (34-42)	40 (35-43)	.04
Domain			
Stress experienced during labor	14 (12-16)	14 (12-16)	.046
Quality of care provision	19 (16-20)	19 (17-20)	.006
Women’s personal attributes	7 (5-8)	7 (5-8)	.45

Abbreviations: BSS-R, Birth Satisfaction Scale–Revised; IQR, interquartile range.

^a^ Scores range from 10 to 50, with higher scores indicating increased satisfaction.

## Discussion

When implemented into standard of care, use of a validated cesarean risk calculator after labor induction was associated with a substantial change in obstetric outcomes. Use of the cesarean risk calculator was associated with a 6% absolute risk reduction in maternal morbidity and improved perception of quality of care. In secondary outcomes, use of the cesarean risk calculator was also associated with a more than 8% absolute risk reduction in cesarean delivery rate.

Data from this prospective cohort study suggest that our improved maternal outcomes may have been secondary to alterations in labor management at the extremes of cesarean delivery risk. With implementation of the calculator into usual care, we demonstrated an associated decrease in actual cesarean delivery rate and complete elimination of cesarean delivery for failed induction in the group with predicted cesarean deliver risk of less than <20.0%. These findings support that, for women at low risk of cesarean delivery using the calculator, patients and clinicians may have been more empowered to achieve a vaginal delivery, thereby avoiding the morbidity associated with a cesarean delivery.

In the group with predicted cesarean delivery risk of 40.0% to 59.9%, although the difference in maternal morbidity was not statistically significant, calculator implementation was associated with a substantial reduction in cesarean delivery rate, which may have contributed to the observed overall associated reduction in maternal morbidity. In the group with predicted cesarean delivery risk of 60.0% or greater, there was no change in cesarean delivery rate. Instead, calculator implementation was associated with a 5-hour shorter time to delivery. These findings support that clinicians may have made the decision for cesarean delivery sooner in women at high risk for cesarean delivery, avoiding the morbidity of a prolonged and ultimately failed induction. Although the reduction in morbidity was not statistically significant, this was likely owing to the small number of women at such high risk for cesarean.

Importantly, use of the calculator and the observed improved maternal outcomes were not associated with increased risk of cesarean delivery or neonatal morbidity, which were of concern to some clinicians at our institution when the calculator was first presented. In fact, calculator implementation was associated with decreased cesarean deliveries. Calculator implementation was also associated with decreased maternal and neonatal length of stay, likely secondary to the decreased cesarean delivery rate.

Numerous prior studies have sought to develop risk prediction calculators in obstetrics for outcomes such as successful trial of labor after cesarean delivery and peripartum hemorrhage.^12,13,14,15^ Other groups have even developed similar calculators to ours for risk of cesarean delivery after labor induction.^16,17^ Such calculators are designed to be used clinically and to have an effect on quality of care. However, work evaluating the effects of implementing such calculators into routine care is scarce. In neonatology, a sepsis risk calculator predicts the likelihood of severe neonatal infection based on factors present at birth. Studies evaluating implementation of the sepsis risk calculator into care have successfully demonstrated that the results alter clinician practice and thereby reduce neonatal antibiotic administration and intensive care unit admissions.^18,19,20^ Here, we demonstrate that a risk prediction calculator within obstetrics is associated with improvements in meaningful perinatal outcomes.

### Strengths and Limitations

Strengths of this study include the prospective evaluation of more than 1500 women. Our high rate of use of the calculator for eligible patients throughout the study period (>80%) likely reflects the success of our multipronged implementation strategy.

Our study was performed at 1 urban academic hospital with exclusion criteria reflecting those used in the calculator’s creation, which may limit generalizability. However, our diverse population is a great advantage to a study focusing on maternal morbidity. In addition, the fact that our institution has a limited number of clinicians with similar practice patterns, and yet the calculator result was still able to have such a significant association, indicates that results might be even stronger in practice models with increased practice variation. An additional limitation is the preimplementation and postimplementation design, susceptible to unmeasured bias. However, no other institutional process changes designed to improve maternal morbidity occurred during the study. Although the results of a large multicenter randomized clinical trial supporting elective induction at a gestational age of 39 weeks may have affected gestational age at induction during our study period,^11^ we were able to control for this difference in our models. Our stratified analysis by predicted cesarean delivery risk is limited by the small sample size at highest risk of cesarean delivery. Finally, although we were able to examine measurable differences in the induction process, such as time to cesarean, the association of knowledge of cesarean delivery risk with decision-making during induction was not evaluated. Such data may provide a deeper understanding of factors mediating the association between implementation of the cesarean risk calculator and reduced obstetric morbidity.

## Conclusions

Clinical use of this validated calculator is a simple intervention. With it, we have the potential to reduce maternal morbidity and cesarean rate broadly, without compromising neonatal outcomes. It is important to note, however, that not every patient with a predicted risk of 60.0% or greater should will require a cesarean delivery. Conversely, even with predicted risk of less than 20.0%, some women will require cesarean delivery. In addition, a result on this validated calculator should never be used to delay a medically indicated induction. The calculator result should always be used in clinical context. Future work should quantitatively and qualitatively evaluate the effect of this intervention on patient and clinician decision-making, as well as examine the effect of the cesarean risk calculator when implemented at diverse sites.
